# Bisignate Surface-Enhanced Raman Optical Activity with Analyte-Capped Colloids

**DOI:** 10.1021/acsnano.4c19027

**Published:** 2025-03-07

**Authors:** Moumita Das, Debraj Gangopadhyay, Valery Andrushchenko, Josef Kapitán, Petr Bouř

**Affiliations:** a Institute of Organic Chemistry and Biochemistry, Academy of Sciences, Flemingovo náměstí 2, Prague 16610, Czech Republic; b Department of Analytical Chemistry, University of Chemistry and Technology, Technická 5, Prague 16628, Czech Republic; c Department of Optics, Palacký University Olomouc, 17. listopadu 12, Olomouc 77146, Czech Republic

**Keywords:** chirality, surface-enhanced Raman optical activity, silver nanoparticles, electronic circular dichroism, chiral analyte capped colloid

## Abstract

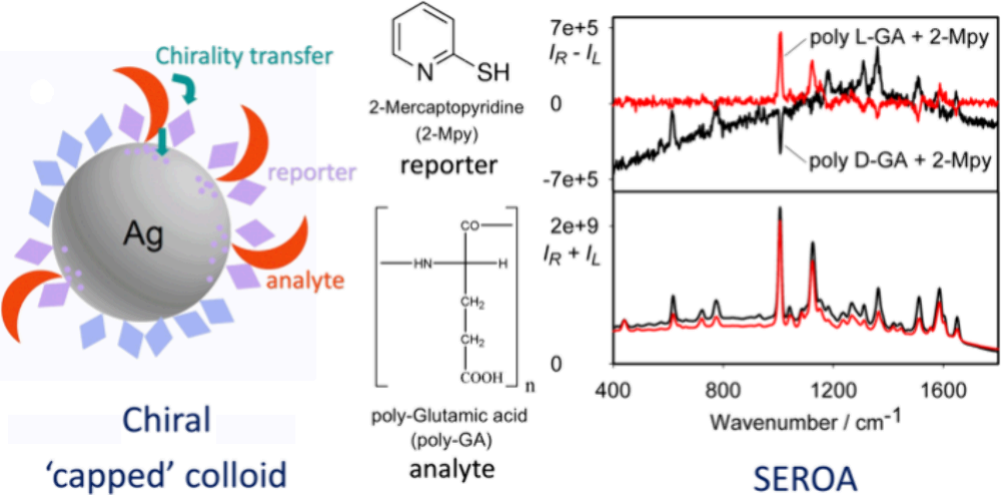

Spectroscopic detection of chiral compounds is often hampered by a low sensitivity. For Raman optical activity (ROA), the signal can be dramatically increased in surface-enhanced experiments. So far, however, reproducible surface-enhanced ROA (SEROA) spectra were obtained for a reporter molecule only via induced chirality, and the intensities were just proportional to the Raman scattering. In the present study, we show that the signal can be substantially increased if colloidal silver nanoparticles are prepared already in the presence of a chiral analyte. In this case, both the analyte’s and reporter’s bands are visible. In addition, some experiments provided bisignate SEROA patterns, thus significantly enhancing information about the molecular structure provided by this spectroscopic method. Increased electronic circular dichroism (ECD) of the capped aggregated colloids suggests that ECD and polarized Raman scattering (ECD–Raman) contribute to the monosignate SEROA intensities, while well-dispersed nonaggregating colloids are important for observation of true (bisignate) molecular vibrational SEROA.

## Introduction

Surface-enhanced Raman optical activity (SEROA) experiments attract attention because of the possibility to detect and study chiral molecules at very low concentrations.^1−8^ In addition, the difference in scattering intensities of right- and left-circularly polarized light provides enhanced information about the absolute configuration and structure of studied molecules. Researchers commonly use silver, gold, or multicomponent nanoparticles, mostly as colloidal suspensions, as substrates to produce significant Raman signal enhancement due to their strong plasmonic properties.^9−18^ Although the technique holds immense potential, it faces numerous challenges regarding reproducibility and interpretation.^5,19−21^ The SEROA component (*I*_R_*– I*_L_) is typically three to five orders of magnitude smaller than the total surface-enhanced Raman scattering (*I*_R_ + *I*_L_, SERS) signal.^16,17,22^ This results in longer accumulation times and a need of colloidal suspensions sufficiently stable in the laser beam.^14,17,23^ The colloid instability is particularly problematic for silver, which is otherwise a very convenient SERS and SEROA substrate, giving large enhancements and resonating at the common 532 nm laser wavelength. The sizes and shapes of the nanoparticles can be sometimes modified to give stable SEROA signals.^24^ As another obstacle, the metallic nanoparticles may change the polarization of light and thus change or destroy the SEROA signal.^21,25^

The interpretation of the SEROA experiment is hampered by the lack of understanding of the interactions between chiral molecules and the metal surface and of the link between the spectral signal and the structure. The orientation and distance of a molecule to the nanoparticle surface have a significant impact on the SERS and SEROA signals, but controlling these parameters is difficult.^13,18,20^

Typically, the colloids in solution are stabilized by an ionic shell, hindering the analyte’s access to the surface. The experimental protocol presented below removes this obstacle and partially improves the reproducibility of the spectra. We prepared “capped” silver nanoparticles, i.e., the colloid is formed already in the presence of the analyte. It appears that the chiral molecule can also stabilize the nanoparticles in the same way as the ions, preventing the aggregation and degradation of SEROA.

On the colloids, molecular chirality is combined with the surface chirality.^26,27^ Quite often, chirality induction occurs, either from chiral molecules to a nonchiral surface or from a chiral surface to nonchiral molecules. Achiral molecules become locally chiral when adsorbed on a metal surface.^26^ In a previous study,^19^ we utilized the “sergeant and soldiers” principle to obtain reproducible, mirror-image SEROA spectra of achiral “reporter” molecules (e.g., mercaptopyridine) and detected chirality induced by chiral “modifiers” (e.g., tartaric acid) at concentrations as low as 10 μM. The chirality was explained by molecular vibrational resonance Raman optical activity,^28^ where the excitation wavelength is close to that of an electronic transition of the system molecule colloid. In a more recent study, Lee et al. conducted similar experiments and measured SEROA spectra on a single-particle substrate.^29^

These “sergeant and soldiers” samples did not exhibit measurable electronic circular dichroism (ECD). However, as pointed out in several studies,^30−32^ ECD in combination with circularly polarized Raman scattering (ECD–Raman effect) can often contribute to the *I*_R_*– I*_L_ intensities of light-absorbing samples. “True” SEROA can then be obtained when the ECD–Raman component is subtracted.^30^

**Figure 1:**
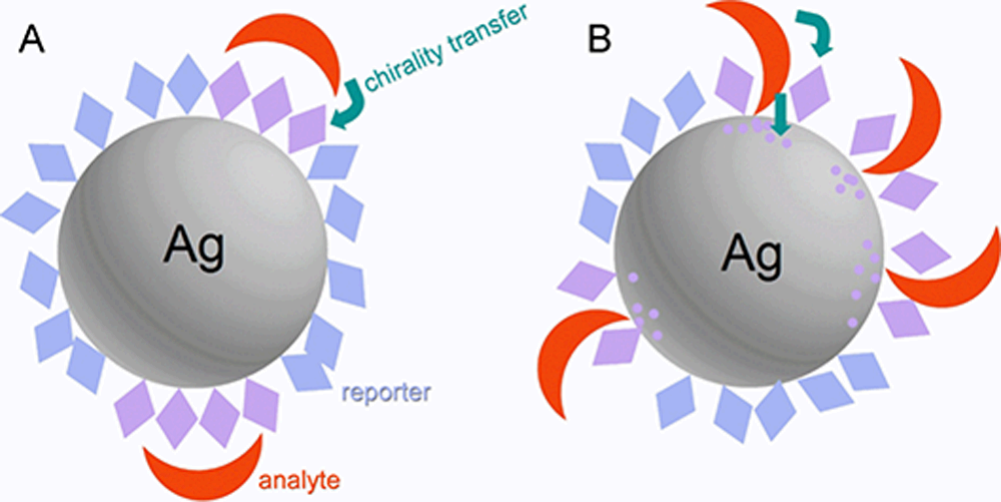
(A) Pure “sergeant and soldiers” experiment where the chirality is induced in the reporter molecules and (B) present protocol where “capped” analytes are closer to the colloid surface.

**Figure 2:**
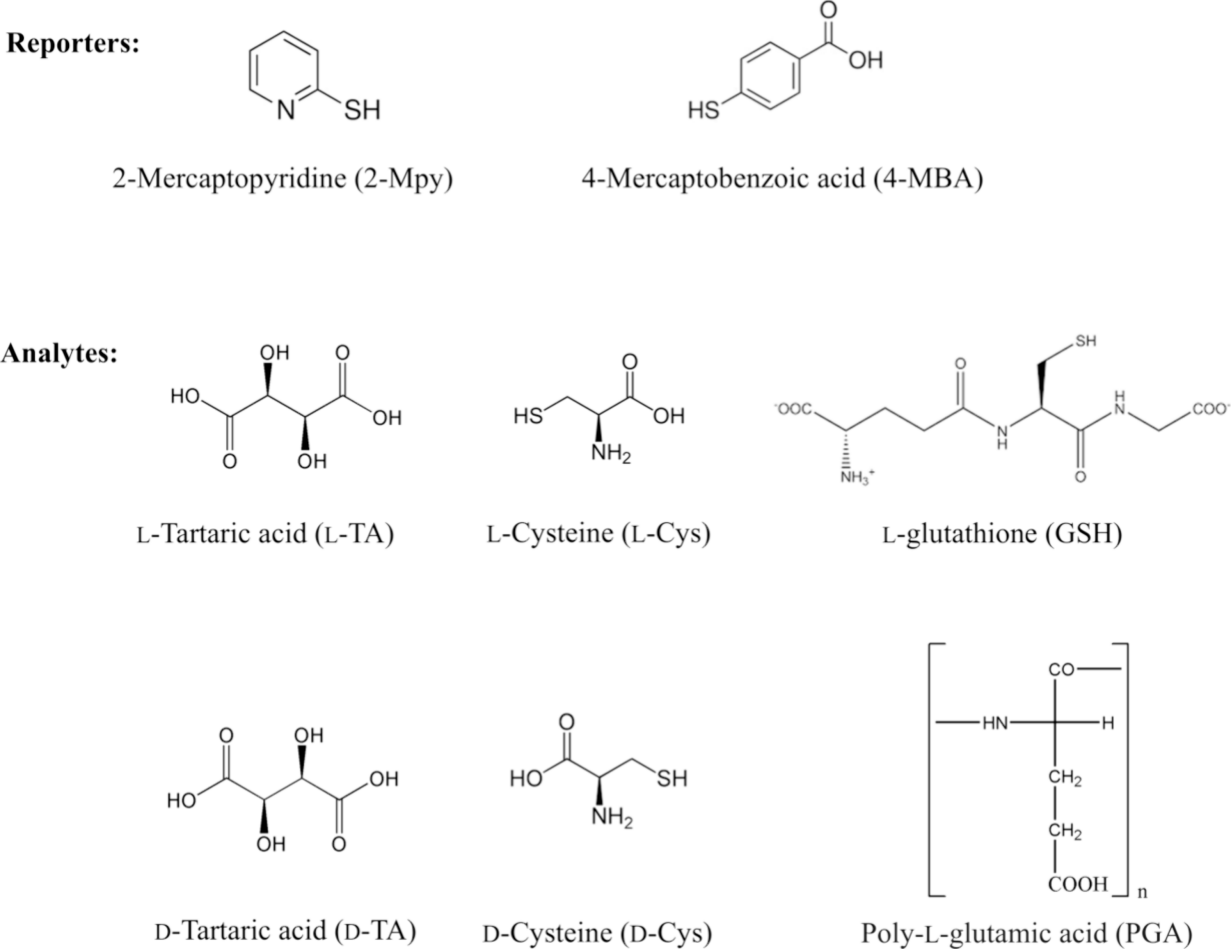
Structures of reporters and analyte molecules used in this study.

The “sergeant and soldiers” and “capped” protocols are explained in a simplified way in Figure 1. The capped silver nanoparticles investigated below are prepared already in the presence of the chiral analyte; however, a small amount of a reporter molecule is still added for signal amplification. The reporter and analyte molecules tested are listed in Figure 2. The applied protocol leads to different spectroscopic properties of the samples. ECD becomes measurable, indicating that the analyte interacts more strongly with the silver surface and that the ECD–Raman effect may also contribute to ROA. The SEROA signal is stronger, and both the reporter’s and the analyte’s SERS bands are observable. In addition, we observe bisignate SEROA bands, verifiable by the “mirror-image” signal for enantiomers. Thus, more information about the structure is encoded in the SEROA spectra, since they are not just proportional to the SERS signal as in the previous experiments.^19^

## Results and Discussion

### Capped vs Bare Colloids

**Figure 3:**
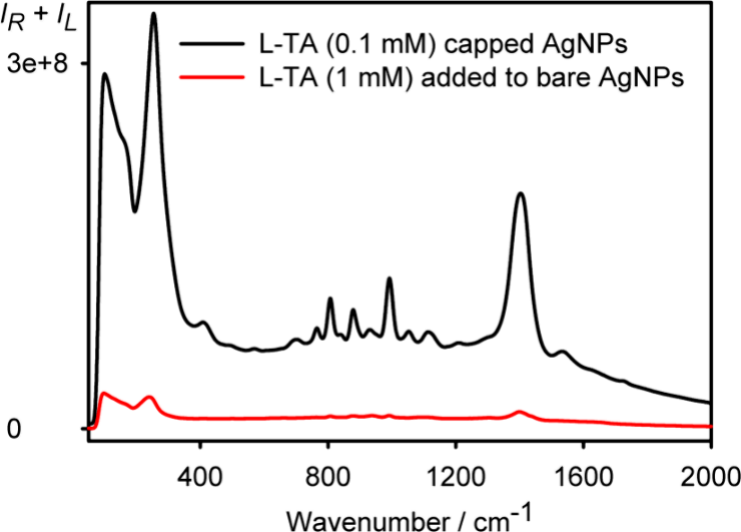
SERS spectra of l-TA obtained with capped and bare colloids.

Compounds such as tartaric acid (TA) with a low affinity toward the silver substrate typically provide very weak SERS.^19,33^ This is because even bare noncapped colloids may still have various chemicals adsorbed on the surface, blocking the interaction with the analyte. However, the capped colloids provide significantly stronger signals, even for a lower concentration, as shown in Figure 3 for TA. In addition, it acts in the same way as the citrate remaining on the surface, stabilizing the colloids and preventing aggregation^34,35^ Indeed, both the borohydride and citrate colloids capped with TA are stable for a long time. Due to this added stability, we can suppose that the spectra come from dispersed nanoparticles rather than from aggregates. The other two analytes, Cys and GSH, bind even stronger to the colloid, via the thiol group,^36,37,49^ preventing aggregation and making the colloid more stable as well.

### Electronic and Vibrational Chirality of Analyte-Capped Colloids

**Figure 4:**
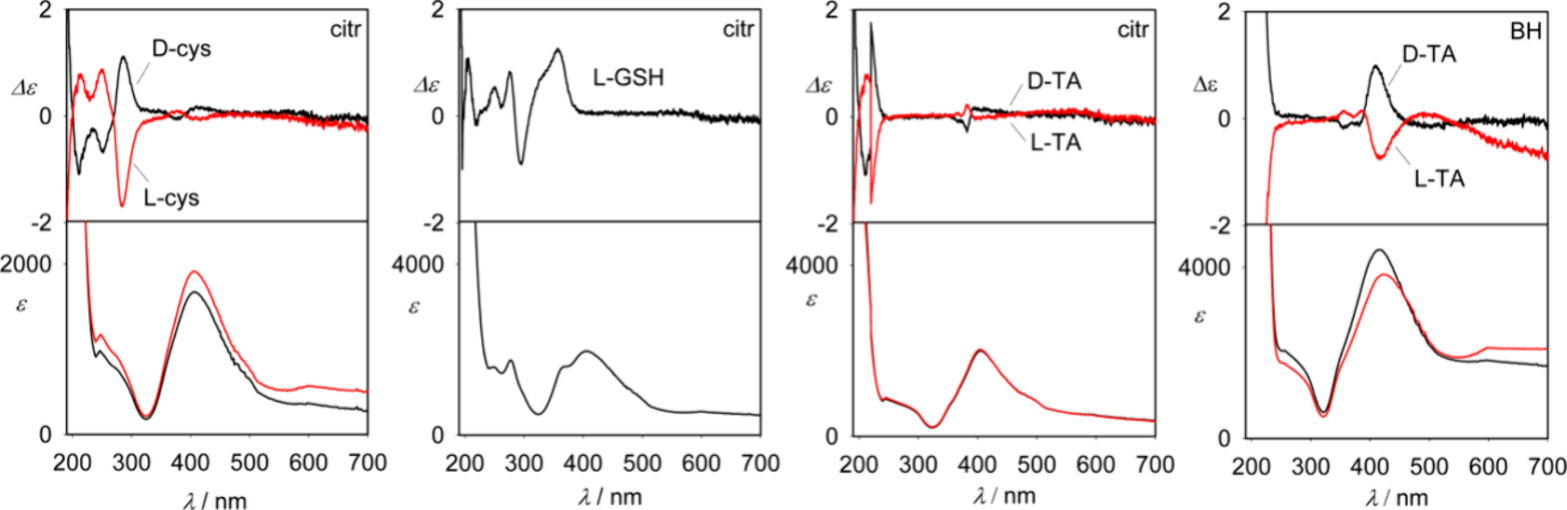
Absorption and ECD spectra of four analytes mixed with citrate (citr) and borohydride (BH) colloids.

Unlike noncapped colloids (Figure S1), the capped nanoparticles exhibit a measurable ECD signal, even at the plasmonic silver band (with a maximum at ∼419 nm, Figure 4). The sulfur-containing analytes (Cys/GSH) gave a richer ECD pattern, having more peaks than TA, presumably because of the mixing of the nanoparticle and analyte electronic states. At the 532 nm excitation wavelength, ECD is immeasurable, except for the Ag_BH_NPs/TA case, where the signal is slightly positive for l-TA (Δε ∼ 0.1 L·mol^–1^·cm^–1^). This corresponds to Kuhn’s dissymmetry parameter (ECD/absorption ratio) of *g* ∼ 5 × 10^–5^.

**Figure 5:**
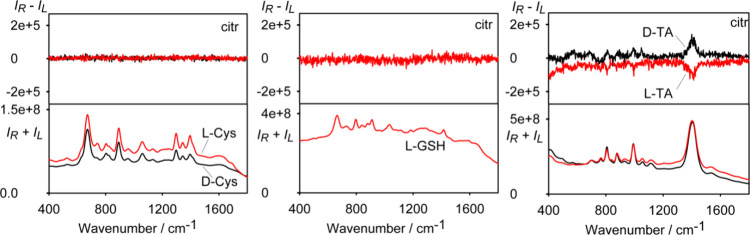
SERS and SEROA spectra of Cys-, GSH-, and TA-capped citrate colloids.

A measurable ROA signal could also be obtained only for the Ag_citr_NPs/TA sample, where the two enantiomers give an opposite sign of otherwise monosignate spectra (Figure 5). In this case, the normalized circular intensity difference (ROA/Raman ratio) is CID ∼ 5 × 10^–4^ at 1400 cm^–1^.

Based on ECD spectra, we can estimate part of SEROA coming from the ECD–Raman effect.^30−32^ We use the simplified formula when the relevant part of SEROA signal is^30^
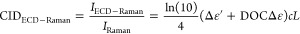
where Δε and Δε′ are ECD intensities for the excitation and scattered light, DOC is the degree of circularity, *c* is the concentration, *L* is the optical path length, and *I*_Raman_ is the Raman intensity. Since Δε = Δε_532_ < 0.1, Δε ∼ 0, *c* = 3.3 × 10^–4^ M, and *L* ∼ 0.1 cm, we obtain CID < 2 × 10^–6^, quite a negligible contribution, which suggests that SEROA of TA primarily comes from resonance ROA scattering.^19^

### Analyte-Capped Colloids with Added Reporters

**Figure 6:**
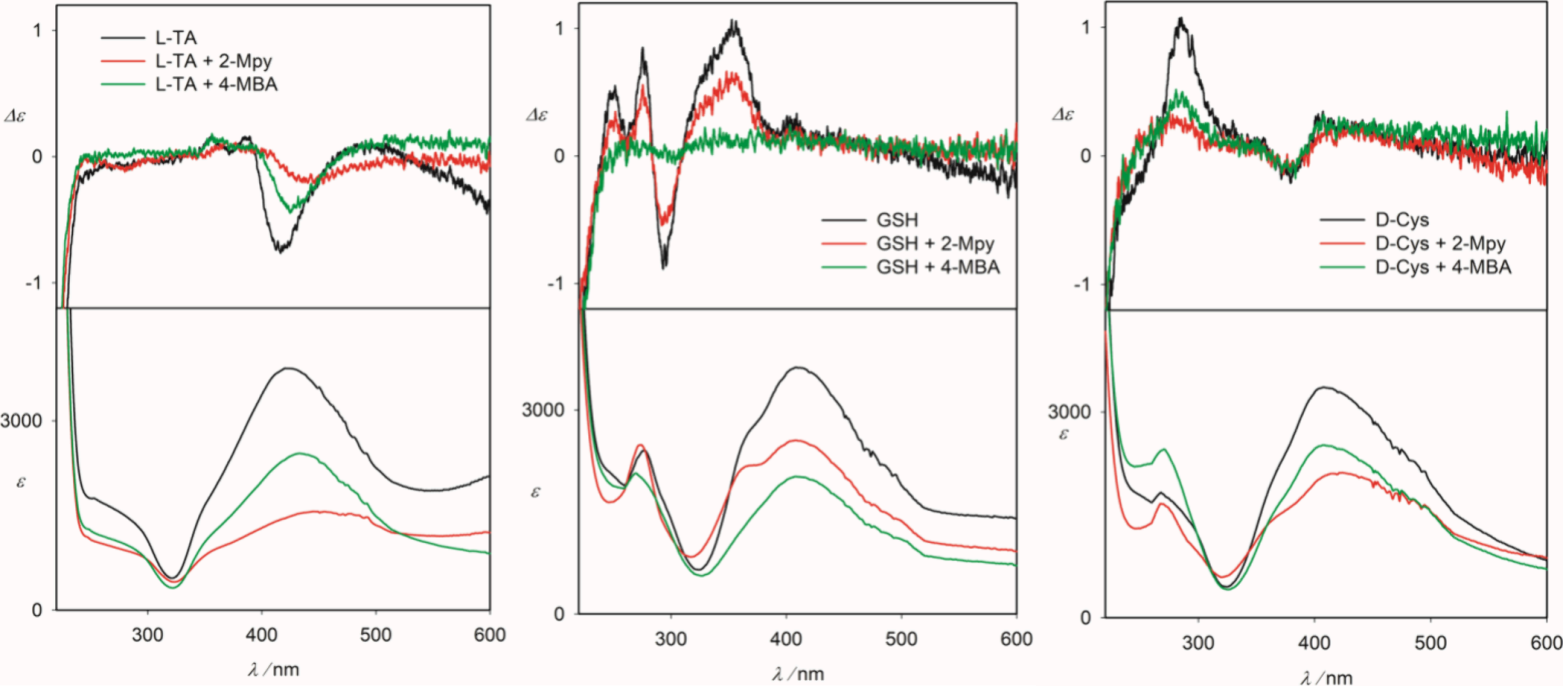
ECD and absorption spectra of l-TA-capped, GSH-capped, and d-Cys-capped colloids without and with 2-Mpy and 4-MBA added.

In this case, the colloids were grown in the presence of the analyte (TA or GSH or Cys), with 2-Mpy and 4-MBA reporter molecules added just before the measurement. This significantly weakens the ECD and absorption spectra (Figure 6), suggesting a partial replacement of the analyte by the 2-Mpy/4-MBA reporters at the silver surface. For TA, the addition of 2-Mpy seems to have a bigger effect on ECD than 4-MBA, while for GSH, 2-Mpy does not destroy the original ECD significantly, while 4-MBA does. For Cys, disruptions by 2-Mpy and 4-MBA are almost comparable.

As discussed previously, additional agents can also modify the protective surface charge of the colloids and cause a partial aggregation into small clusters.^38,39^ The partial aggregation and close binding to the silver surface are favorable conditions for a strong SERS signal, especially when a molecule occurs in “hot spots” of increased electromagnetic field intensity.^40^

**Figure 7:**
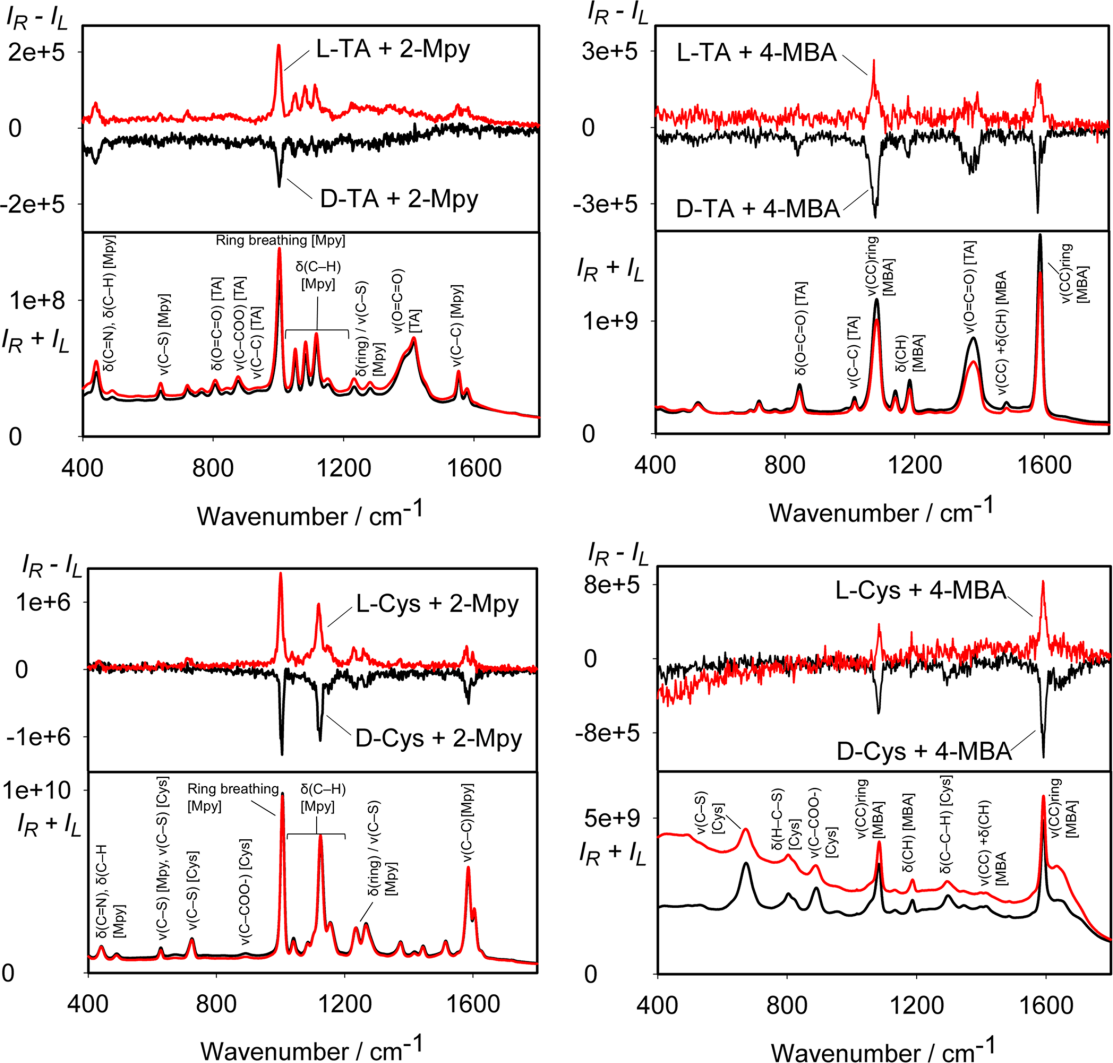
SERROA and SERRS spectra of analyte-capped colloids after the addition of reporter molecules.

Indeed, the reporter-treated capped colloids exhibited strong SERS. For TA and cysteine, the signal was accompanied by SEROA (Figure 7). For glutathione, we still could not get a measurable SEROA signal. The bigger SERS signal can also be related to a combination of the plasmon and molecular resonance.^38,39,41,42^ A monosignate ROA pattern has also been observed under resonance in previous studies.^32,43^ For TA, the SERRS and SERROA bands come from both the chiral analytes and the achiral reporters, while for cysteine, the SERROA spectrum contains only reporter bands. For example, the bands at 1396 and 1380 cm^–1^ observed for TA-capped colloid in the presence of 2-Mpy and 4-MBA, respectively, are due to the symmetric stretching (O=C=O) vibration of TA (Table S1). The CID ratio at 1380 cm^–1^ is about 2.5 × 10^–4^ for the TA/4-MBA system. For the cysteine-capped colloid, bands at 889 and 667 cm^–1^ (both with 2-Mpy and 4-MBA) correspond to the C–COO– stretching and C–S stretching of the Cys molecule (Table S2); other bands correspond to vibrations of 2-Mpy or 4-MBA (Tables S3 and S4). We can conclude that in these systems, the SERS signal of the analyte bands is weaker, but the chirality is amplified through the interaction with the reporters. Pertaining to the “sergeant and soldiers” principle, the reporter plays the key role in producing mirror image SEROA spectra for the two enantiomers of the analyte.

### Colloids Capped Both with 2-Mpy Reporter and TA Analyte

**Figure 8:**
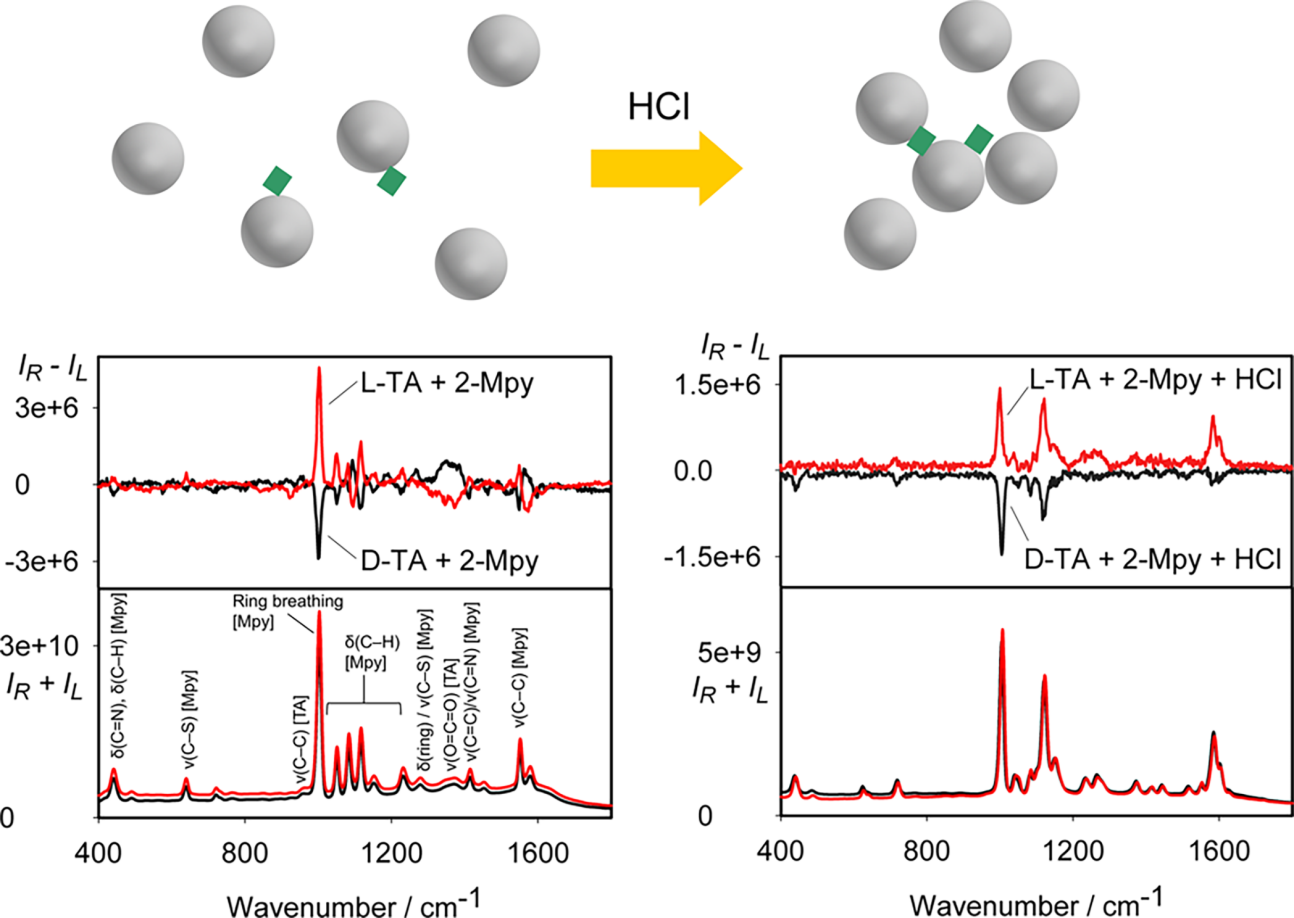
SEROA and SERS spectra of TA- and 2-Mpy-capped colloids before (left) and after (right) the addition of HCl. Schematics illustrating the system aggregation after addition of HCl is shown on top.

These colloids were grown in the presence of both TA and 2-Mpy simultaneously and are probably the most interesting because of the bisignate SEROA spectra for TA and 2-Mpy (Figure 8, ECD in Figure S2). For other reporters and analytes, the samples were not stable during the measurement. The “mirror-image” spectra of the enantiomers confirm that the signal does come from the analyte and not from instrumental artifacts. Apparently, the reporter and analyte binding to the silver is quite strong, allowing for a long (∼20 h) SEROA spectral accumulation (Figure S3). We optimized the TA to 2-Mpy ratio, ensuring that analytes bind to the silver surface while reporters bind minimally, preserving the desired spectral properties (Figure S4). We see SERS bands of 2-Mpy but also of TA, such as those at 1400, 992, 930, 880, 843, and 806 cm^–1^, corresponding to the ν_s_(O–C–O), ν(C–C), ν(C–COO), and δ(O–C–O) vibrations of TA molecule (ν_s_ = symmetric stretching; ν = stretching; δ = bending, Tables S1 and S3). The SEROA spectral pattern has been verified by independent measurements on different samples on both the BioTools and Zebr ROA spectrometers (Figure 8 and Figure S5, respectively).

Due to the stabilized attachment of the reporter (2-Mpy) and analyte (TA), a well-dispersed nonaggregated colloid was produced and bisignate SEROA was measured. When a small amount of HCl (1 μL of 0.5 M) is added, an aggregation is triggered due to acidic pH (2.5), and monosignate mirror-image SEROA spectra are produced (Figure 8). This can be connected to the change of resonance conditions and directional polarization prevailing in aggregated colloids.^24^

To obtain further insight into these observations, we measured Raman and ROA spectra of solutions of TA and 2-Mpy in water without the colloids. Here, no interaction leading to significant spectral changes has been observed, indicating the importance of the silver surface and surface chirality for the chirality transfer (Figure S6).

Mechanistically, when nanoparticles are grown in the presence of a chiral analyte and a small amount of an achiral reporter molecule, spectral bands corresponding to both species emerge. In the first step, the chiral analyte forms a supramolecular assembly with a specific orientation dictated by its chirality. In the second step, the addition of the achiral reporter molecule, a strong SERS scatterer with a thiol group, leads to its binding to the metal surface, and chirality is induced by the analyte–metal interface. Since most of the surface is occupied by the analyte and only a limited amount of the reporter is present, the reporter molecule generates hot-spot regions, enhancing the overall signal of the nanoparticle–analyte–reporter supramolecular assembly.

### Computational Models

The spectroscopic characteristics of the analyte and reporter, both concurrently capped and chirally organized on the silver surface, have been investigated using clusters consisting of the analyte (l-TA/d-TA) only (Figure S7) along with the reporter ion (2-Mpy) (Figures S8 and S9) on the surface of 16 or 7 silver atoms. SERS and SEROA spectra were simulated for five excitation frequencies (532, 534, 540, 522, and 467 nm). The calculated SEROA spectra at 532 and 534 nm excitations show a close resemblance to the experimental SEROA spectra, measured at 532 nm excitation, while the spectra calculated at other excitation wavelengths show no resemblance to the experimental spectra, which confirms the importance of resonance for the SERS and SEROA phenomena. The computations also helped us to verify the band assignments (Tables S1–S5).

### Colloid Stabilization Using Capping

**Figure 9:**
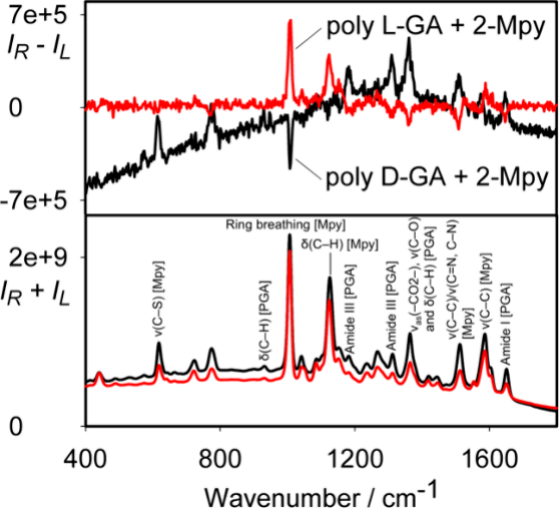
SEROA and SERS spectra of PGA and 2-Mpy at low concentrations.

The capped colloids discussed so far were stabilized using 1% poly(vinyl alcohol) (PVA), added at the boiling stage. To explore the idea of colloid modification at the initial stage, we prepared a colloid capped and stabilized with polyglutamic acid (PGA) alongside 2-Mpy, without using PVA. As apparent in Figure 9, bisignate SEROA appears already here, although for d-PGA the baseline is distorted, and the d and l enantiomers provide opposite spectra only approximately. This can be related to different molar mass distributions and purities of the commercially available PGA.^44,45^ The colloids remained stable for ∼1.5 h, which was enough to accumulate an SEROA signal (Figure S3), although for further reduction of the noise, much longer times would be desirable.^46^ As in the previous cases, a mixture of PGA and 2-Mpy bands is observable. The bands at 1674, 1563, 1409, 1442, 1251–1304, 1041, and 950 cm^–1^ correspond to the amide I, ν_as_(−CO_2_−), amide II, symmetric δ(CH_2_), ν(C–O) and δ(C–H), and amide III, wagging and rocking δ(C–H_2_), localized δ(C–H), and out-of-plane vibrational modes of PGA (Table S5).

## Conclusions

In conclusion, we developed an experimental protocol involving “capping” the silver colloid with chiral analytes in the preparatory stage, which appeared viable for the sensitive detection of model chiral organic molecules at low concentrations using chiral SERS spectroscopy. In the SEROA spectra thus obtained, both the analyte and reporter bands were distinguishable. Additionally, electronic circular dichroism spectroscopy indicated a significant interaction of the analytes attached to the colloid with silver plasmonic excitations. A relatively large ECD of the capped colloid suggested a significant role of electronic circular dichroism in the measured SEROA signal. The monosignate character of the spectra from aggregated colloids is consistent with a resonance ROA enhancement and the single electronic state theory. At low concentrations and for stabilized nonaggregated colloids, however, bisignate SEROA appeared, suggesting a more complicated origin of the signal, which was destroyed by induced aggregation. However, despite being extremely sensitive, informative, and stable, the reproducibility of such experiments can still be improved, and further development is needed for more routine analytical use. Nevertheless, we find the SERS chirality detection fascinating and very promising for future detection and studies of biomolecular systems.

## Methods

### Materials

Commercial chemicals (Sigma-Aldrich) and Milli-Q water were used throughout.

### Synthesis of “Capped” Borohydride Colloids

Borohydride reduced silver nanoparticles (Ag_BH_NPs) capped with TA were prepared according to Shrivas et al.^47^ In a conical flask holding 2 mL of an ice-chilled 2 mM NaBH_4_ solution, a mixture of solutions (15 mL of 3 mM TA and 1 mL of 0.1 M NaOH) was added under steady stirring. After that, 15 mL of AgNO_3_ solution (1 mM) was added to the flask and agitated for 1 h at room temperature. The color of the reaction mixture changed from colorless to bright yellow, indicating that AgNPs capped with TA were formed.

### Synthesis of “Seed” Citrate Colloids

The preparation of citrate reduced silver nanoparticles (Ag_citr_NPs) also followed previous works.^48−50^ Shortly, 9.3 mg of AgNO_3_ was dissolved in 100 mL of boiling water, and 1% trisodium citrate dihydrate solution (1 mL) and 5 mL of 1% poly(vinyl alcohol) (PVA) were added one after the other to the boiling solution. The solution was boiled for 45 min, during which it changed from colorless to greenish yellow/brown. As the reaction vessel was cooled to room temperature, the product looked greenish yellow in reflected light and reddish in transmitted light.

### Preparation of “Capped” Citrate Colloids

Aqueous solutions of the capping agents (l- and d-cysteine, l- and d-tartaric acid, and l-glutathione, 1–0.1 mM) were prepared, and 1 mL of freshly prepared colloidal AgNPs was added to 1 mL of capping agents under constant stirring immediately after synthesis. For capping with both the chiral analyte and achiral reporter molecules, 20 μL of 2-mercaptopyridine (2-Mpy)/4-mercaptobenzoic acid (4-MBA) (0.1 mM) and 1 mL of TA (1 mM) were added to 2 mL of freshly prepared colloid under constant stirring. The surface concentration of the analyte (for TA: ∼3.5 Å^–2^) was much higher than that of the reporter (for 2-Mpy: ∼0.01 Å^–2^), to ensure that analyte molecules adsorb maximally on the surface. The capped colloids were stored in a dark and dry environment for 24 h and then used for spectroscopic studies. The colloids were stored at 4 °C for use in a longer time.

The borohydride-reduced colloids gave stronger ECD with a chiral analyte but weaker and less stable SEROA. Therefore, the citrate-reduced colloids were used in most experiments.

### Measurement of UV–Vis Absorption and ECD Spectra

Absorption and ECD spectra of the analytes were measured for 1 mM aqueous solutions in a 1 mm rectangular quartz cell with a JASCO J-815 spectrometer (JASCO Corporation, Japan). Three scans were averaged, with a 20 nm/min scanning speed and a 4 s response time, within 185–700 nm (high absorption regions were omitted). For the capped silver nanoparticles, the spectra were averaged from nine scans, using 10 nm/min scanning speed and an 8 s response time, within ∼350–500 nm. Absorption and ECD spectra of distilled water measured under identical conditions were subtracted from the sample spectra.

### Measurement of SERS and SEROA Spectra

The colloids with and without capping were used for the measurements about 48 h after preparation. The spectra were collected with either BioTools or Zebr ROA spectrometers, providing a similar performance. A BioTools ChiralRaman-2X spectrometer is operating within 90–2100 cm^–1^ and with a 532 nm excitation laser wavelength. The laser power was adjusted to 100–200 mW at the beginning of the measurement not to saturate the detector. SEROA spectra were accumulated over a longer time (∼22 h) for stable samples and a shorter one (∼4–5 h) for less stable ones. The Zebr spectrometer operates in an extended spectral range,^51^ 50–4500 cm^–1^; here, the laser power was 150 mW and stable SEROA spectra were accumulated for ∼15 h.

### Computational Details

To assign the vibrational bands, Raman and ROA spectra were simulated for simplified systems containing reporters and analytes and a few silver atoms. Within the Gaussian16 program,^52^ the B3LYP^53^ functional was utilized with the 6-311++G** basis set for 2-Mpy and TA and the MWB28 pseudopotential and basis set^54^ for silver. The conductor-like polarizable solvent model (CPCM)^55^ was used to represent the environment. Raman and ROA spectra at the pre-resonance condition were computed by considering the harmonic approximation at several excitation frequencies.

## Supporting Information Available

Absorption and ECD spectra of bare analyte (l-TA), a mixture of analyte and silver colloid, and analyte-capped silver colloid, showing that “capping” with a chiral analyte produces “chiral” colloids (Figure S1); absorption and ECD spectra of capped colloids with TA and 2-Mpy (Figure S2); time evolution of SEROA spectra for silver nanoparticles capped simultaneously with TA and 2-Mpy, and capped simultaneously with poly-GA and 2-Mpy (Figure S3); colloidal systems prepared with different amounts of 2-Mpy just after colloid synthesis and after 24 h, and example of SERS and SEROA spectra at too high and low concentrations (Figure S4); bisignate SEROA and SERS spectra of TA and 2-Mpy capped AgNPs within 50–4000 cm^–1^ (Figure S5); ROA and Raman spectra of TA (1M), its mixture with 2-Mpy (10:1 molar ratio), and Raman spectrum of 2-Mpy (0.1M) (Figure S6); model of l-, d-TA/silver cluster of 16 silver atoms and calculated Raman and SERS spectra at 532 nm excitations showing close resemblance to experimental Raman and SERS spectra, measured with 532 nm excitation (Figure S7); model of the 2-Mpy/ l-, d-TA/silver cluster of 16 silver atoms and calculated SEROA spectra at 532 nm excitations showing close resemblance to the bisignate experimental SEROA spectra, measured with 532 nm excitation (Figure S8); model of 2-Mpy/ l-, d-TA/silver cluster of seven silver atoms and calculated SEROA spectra at 532 and 534 nm excitations showing close resemblance to the bisignate experimental SEROA spectra, measured with 532 nm excitation (Figure S9); vibrational assignment of selected SERS bands of TA (Table S1); vibrational assignment of Cys SERS bands (Table S2); vibrational assignment of 2-Mpy SERS bands (Table S3); vibrational assignment of 4-MBA SERS bands (Table S4); and vibrational assignment of additional SERS bands due to PGA (Table S5) (PDF)
