# Supplementary material for: Bisignate Surface-Enhanced Raman Optical Activity with Analyte-Capped Colloids
Source: ACS Nano. 2025 Mar 7;19(10):10412–20. doi: 10.1021/acsnano.4c19027 (PMC11924305; doi:10.1021/acsnano.4c19027)
Supplement: Supplementary file 1 — nn4c19027_si_001.pdf [file nn4c19027_si_001.pdf]

Supporting Information

Bisignate Surface-Enhanced Raman Optical Activity with Analyte-Capped Colloids

Moumita Das,[a,b] *Debraj Gangopadhyay,*[*,a] *Valery Andrushchenko,*[a] *Josef Kapitán*[c] *and Petr Bouř*[a,b]

[a]Institute of Organic Chemistry and Biochemistry, Academy of Sciences, Flemingovo náměstí 2, Prague 16610, Czech Republic

[b]Department of Analytical Chemistry, University of Chemistry and Technology, Technická 5, Prague 16628, Czech Republic

[c]Department of Optics, Palacký University Olomouc, 17. listopadu 12, Olomouc 77146, Czech Republic

*debraj.gangopadhyay@uochb.cas.cz

Contents

Figure S1. Absorption and ECD spectra of bare analyte (L-TA), a mixture of analyte and silver colloid, and analyte-capped silver colloid, showing that ‘capping’ with a chiral analyte produces ‘chiral’ colloids.

Figure S2. Absorption and ECD spectra of capped colloids with TA and 2-Mpy.

Figure S3. Time evolution of SEROA spectra for silver nanoparticles: (A) capped simultaneously with TA and 2-Mpy, (B) capped simultaneously with poly-GA and 2-Mpy (BioTools ROA spectrometer).

Figure S4. (Top) Colloidal systems prepared with different amounts of 2-Mpy; panel A: just after colloid synthesis and panel B: after 24 hours; red arrow points to the optimal condition. (Bottom) example of SERS and SEROA spectra at too high and low concentrations.

Figure S5. Bisignate SEROA and SERS spectra of TA and 2-Mpy capped AgNPs within 50-4000 cm^{-1} (ZEBR ROA spectrometer).

Figure S6. ROA and Raman spectra of TA (1M), its mixture with 2-Mpy (10:1 molar ratio), and Raman spectrum of 2-Mpy (0.1M).

Figure S7. Model of L-, D-TA/ silver cluster of 16 silver atoms. Calculated Raman and SERS spectra at 532 nm excitations show close resemblance to experimental Raman and SERS spectra, measured with 532 nm excitation.

Figure S8. Model of 2-Mpy/ L-, D-TA/ silver cluster of 16 silver atoms. Calculated SEROA spectra at 532 nm excitations show close resemblance to the bisignate experimental SEROA spectra, measured with 532 nm excitation.

Figure S9. Model of 2-Mpy/ L-, D-TA / silver cluster of 7 silver atoms. Calculated SEROA spectra at 532 nm and 534 nm excitations show close resemblance to the bisignate experimental SEROA spectra, measured with 532 nm excitation.

Table S1. Vibrational assignment of selected SERS bands of TA.

Table S2. Vibrational assignment of Cys SERS bands.

Table S3. Vibrational assignment of 2-Mpy SERS bands.

Table S4. Vibrational assignment of 4-MBA SERS bands.

Table S5. Vibrational assignment of additional SERS bands due to PGA.

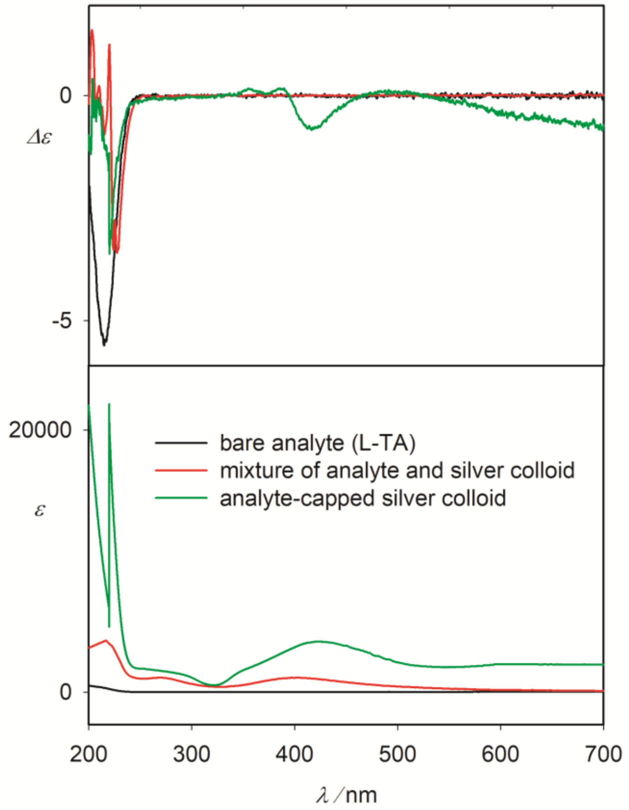

Figure S1. Absorption and ECD spectra of bare analyte (L-TA), a mixture of analyte and silver colloid, and analyte-capped silver colloid, showing that 'capping' with a chiral analyte produces 'chiral' colloids.

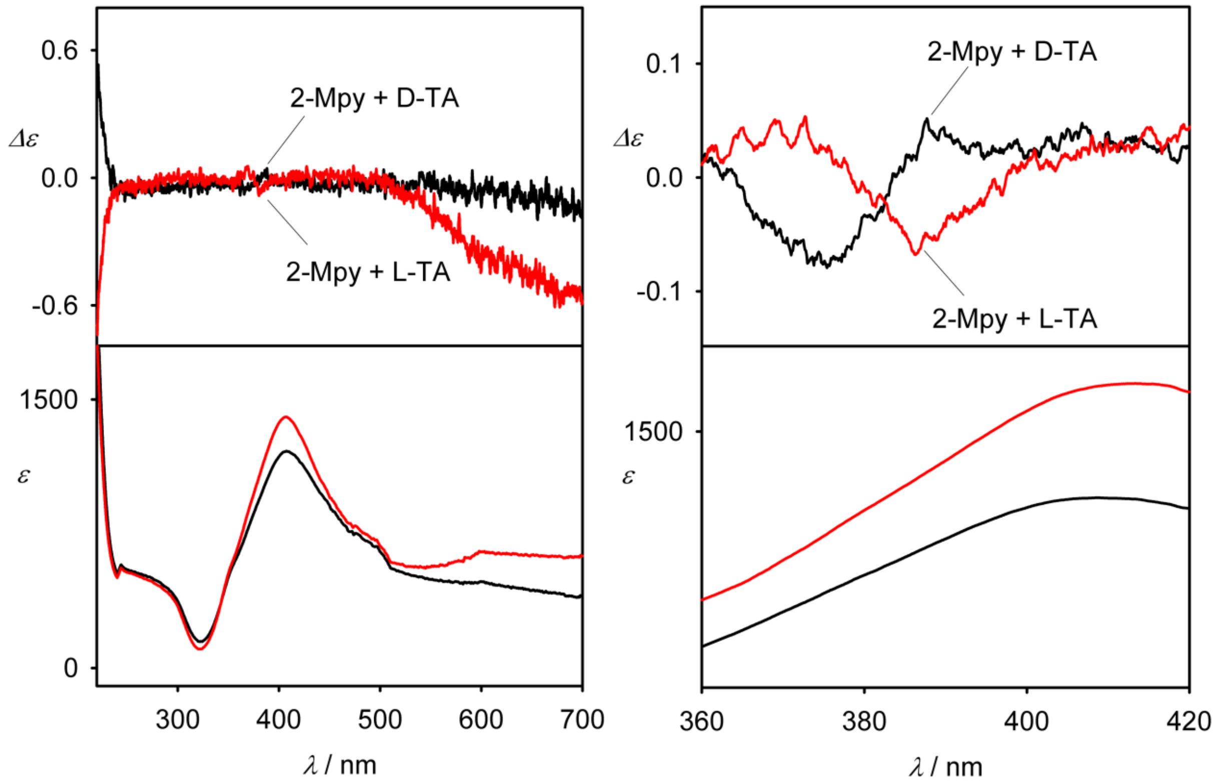

<u>Figure S2.</u> Absorption and ECD spectra of capped colloids with TA and 2-Mpy.

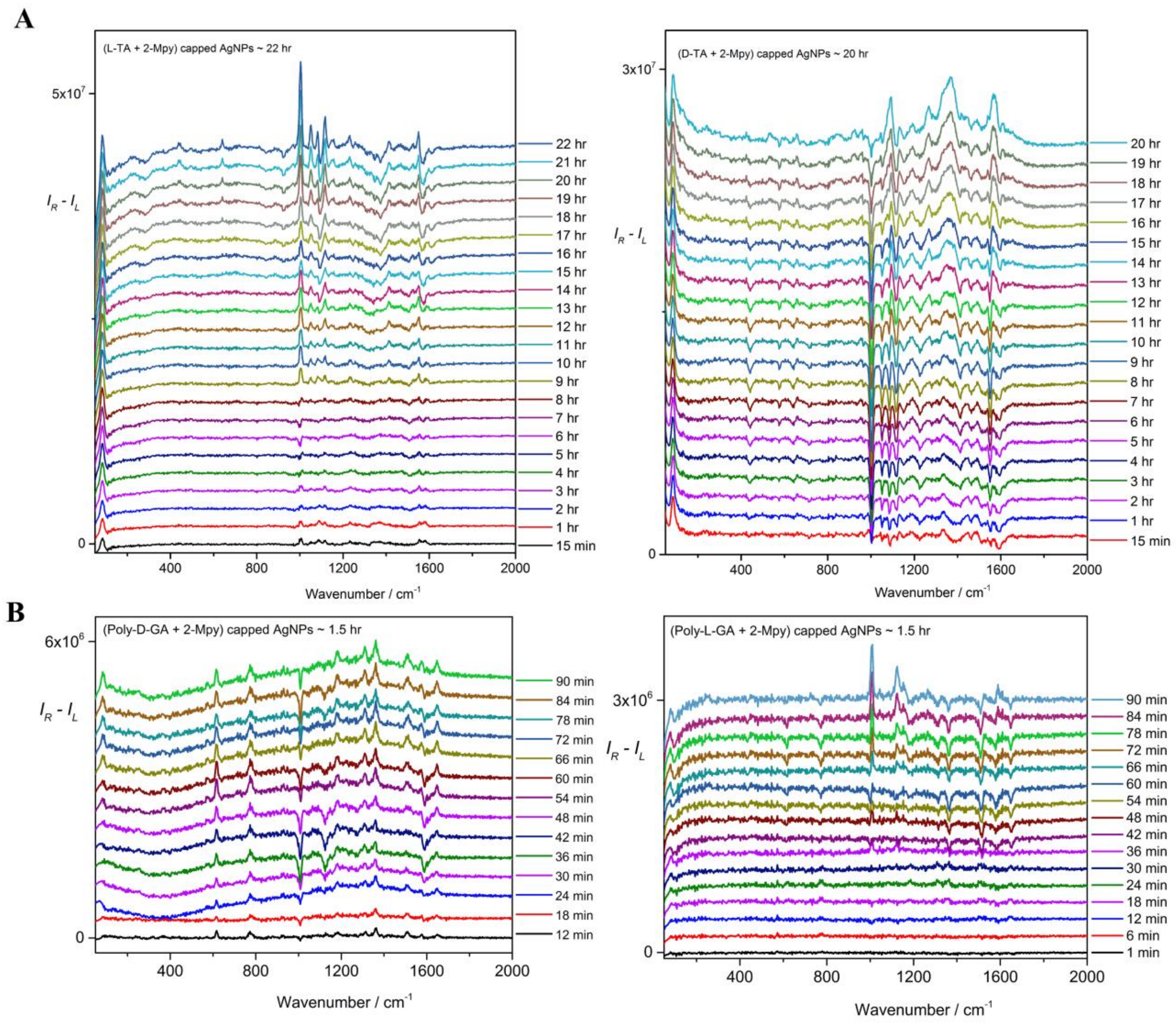

Figure S3. Time evolution of SEROA spectra for silver nanoparticles: (A) capped simultaneously with TA and 2-Mpy, (B) capped simultaneously with poly-GA and 2-Mpy (BioTools ROA spectrometer).

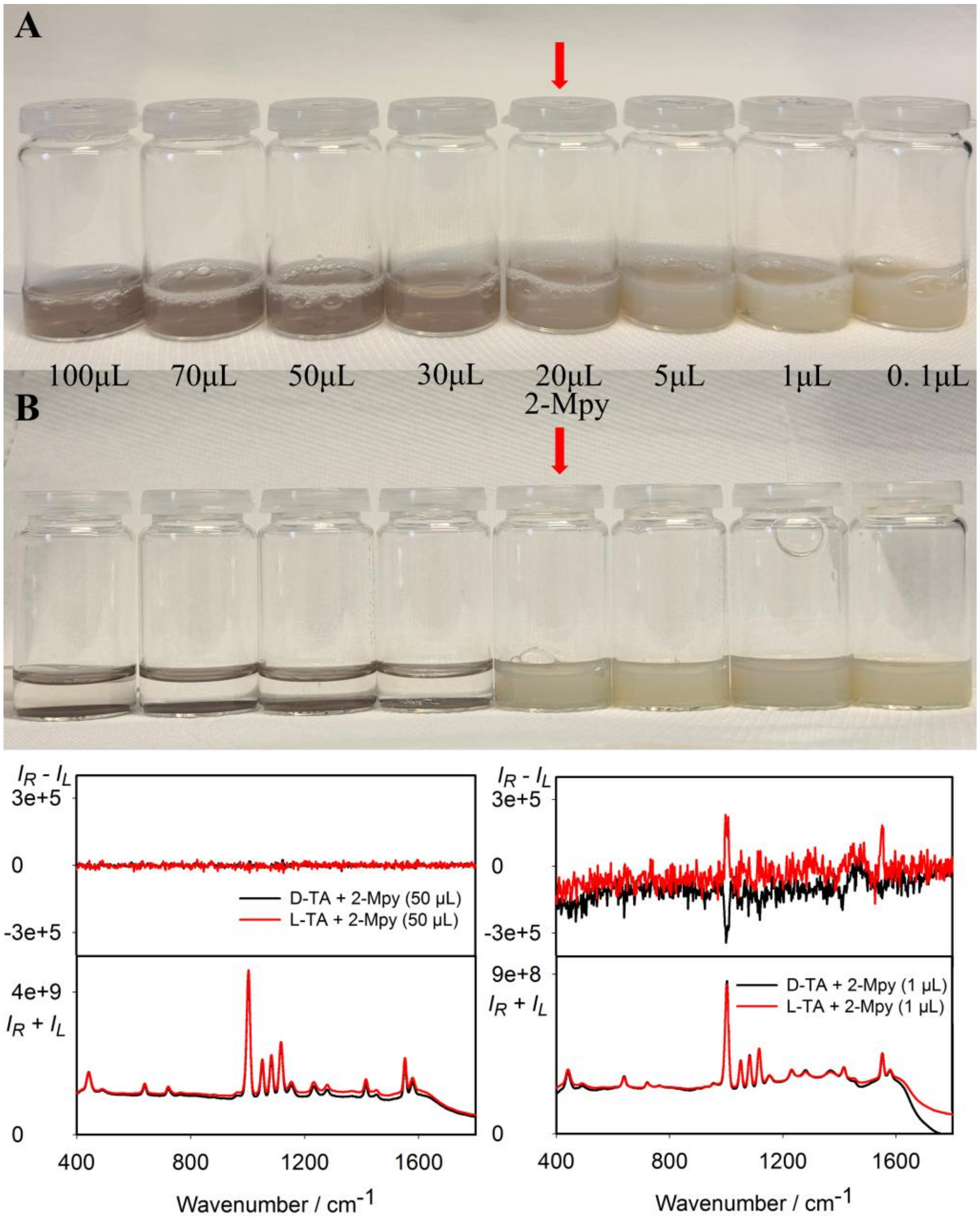

Figure S4. (Top) Colloidal systems prepared with different amounts of 2-Mpy; panel A: just after colloid synthesis and panel B: after 24 hours; red arrow points to the optimal condition. (Bottom) example of SERS and SEROA spectra at too high and low concentrations.

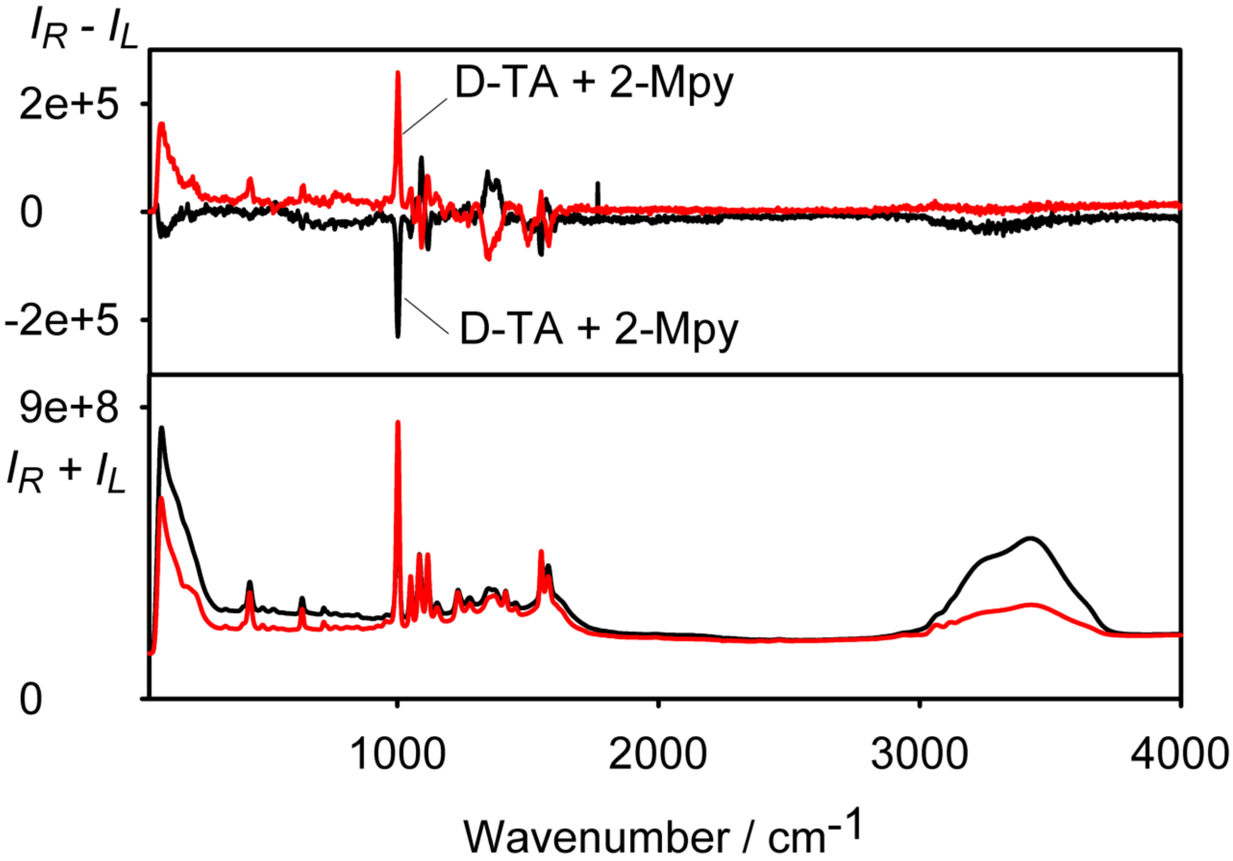

Figure S5. Bisignate SEROA and SERS spectra of TA and 2-Mpy capped AgNPs within 50-4000 cm^{-1} (ZEBR ROA spectrometer).

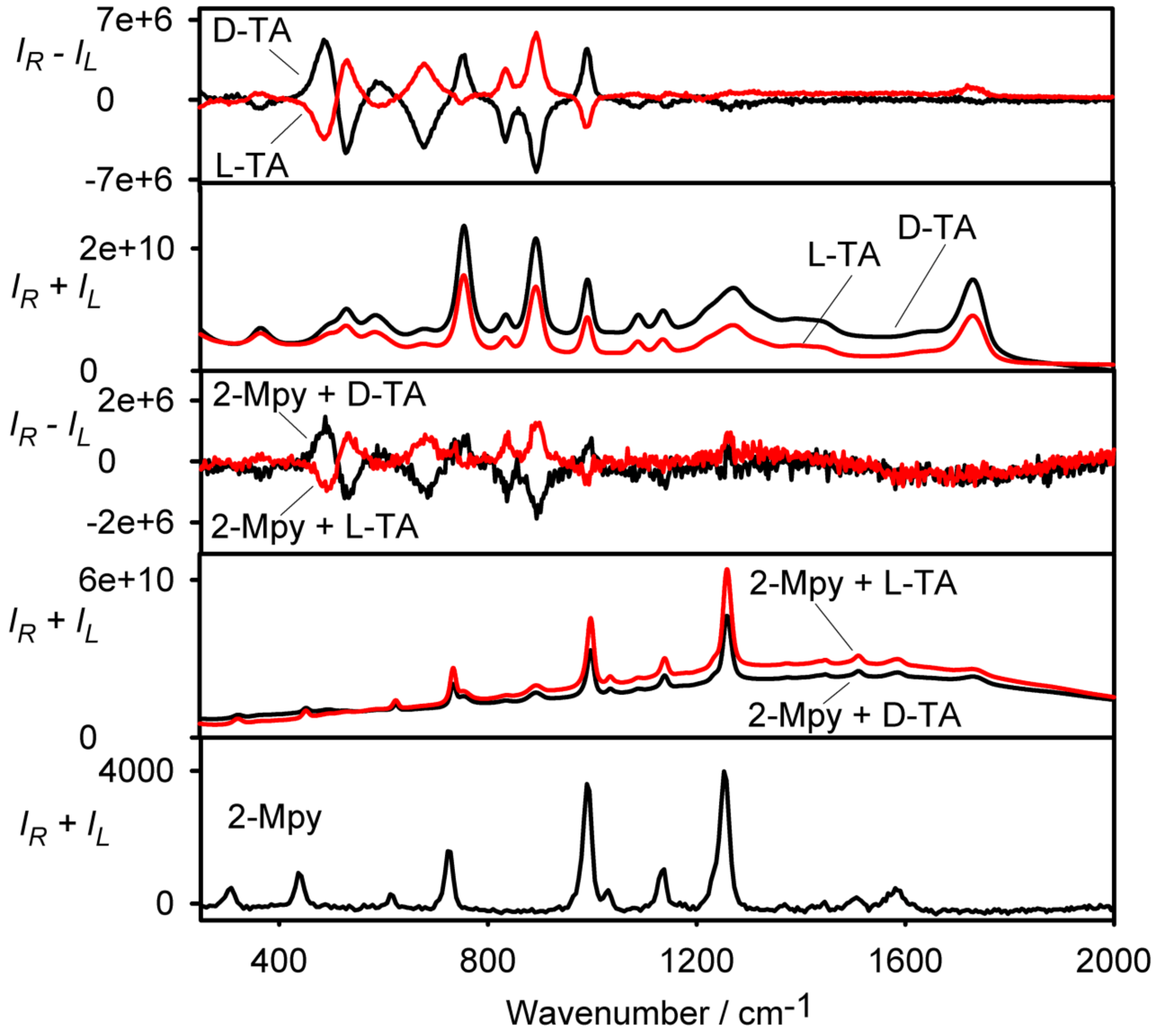

Figure S6. ROA and Raman spectra of TA (1M), its mixture with 2-Mpy (10:1 molar ratio), and Raman spectrum of 2-Mpy (0.1M).

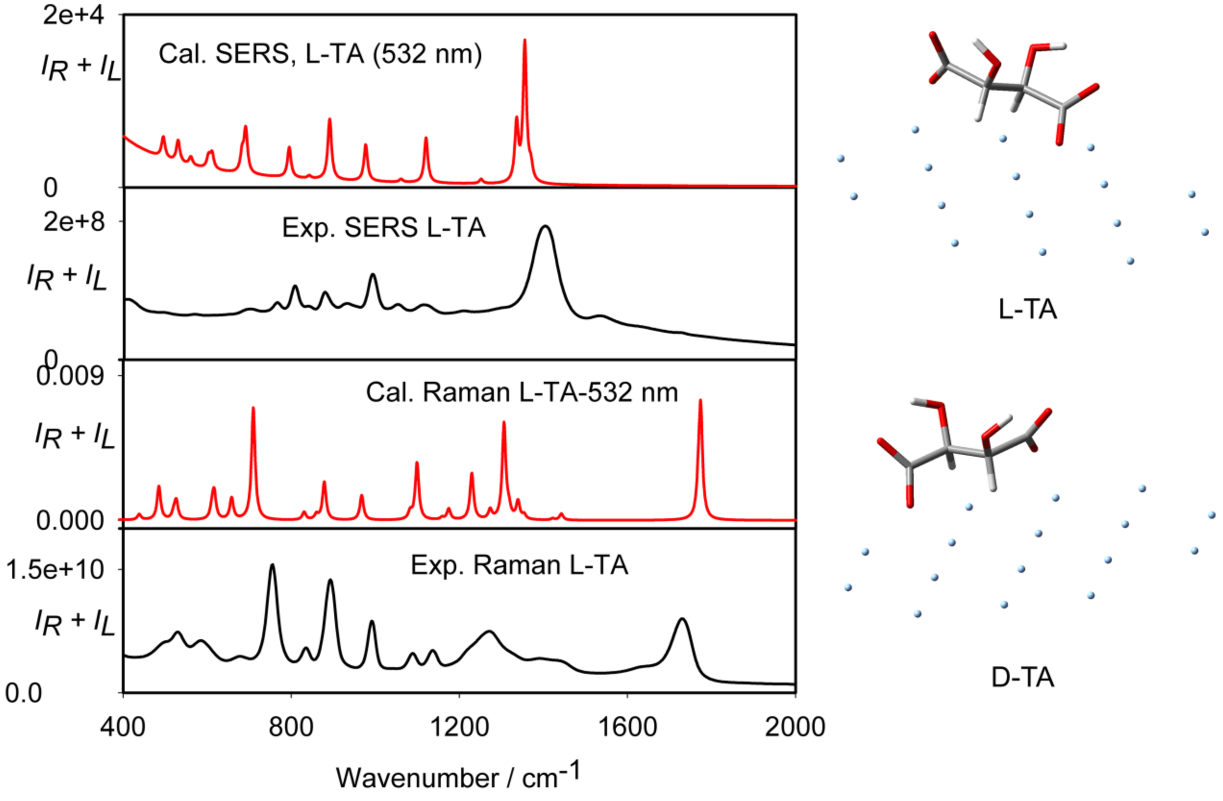

<u>Figure S7</u>. Model of L-, D-TA/ silver cluster of 16 silver atoms. Calculated Raman and SERS spectra at 532 nm excitations show close resemblance to experimental Raman and SERS spectra, measured with 532 nm excitation.

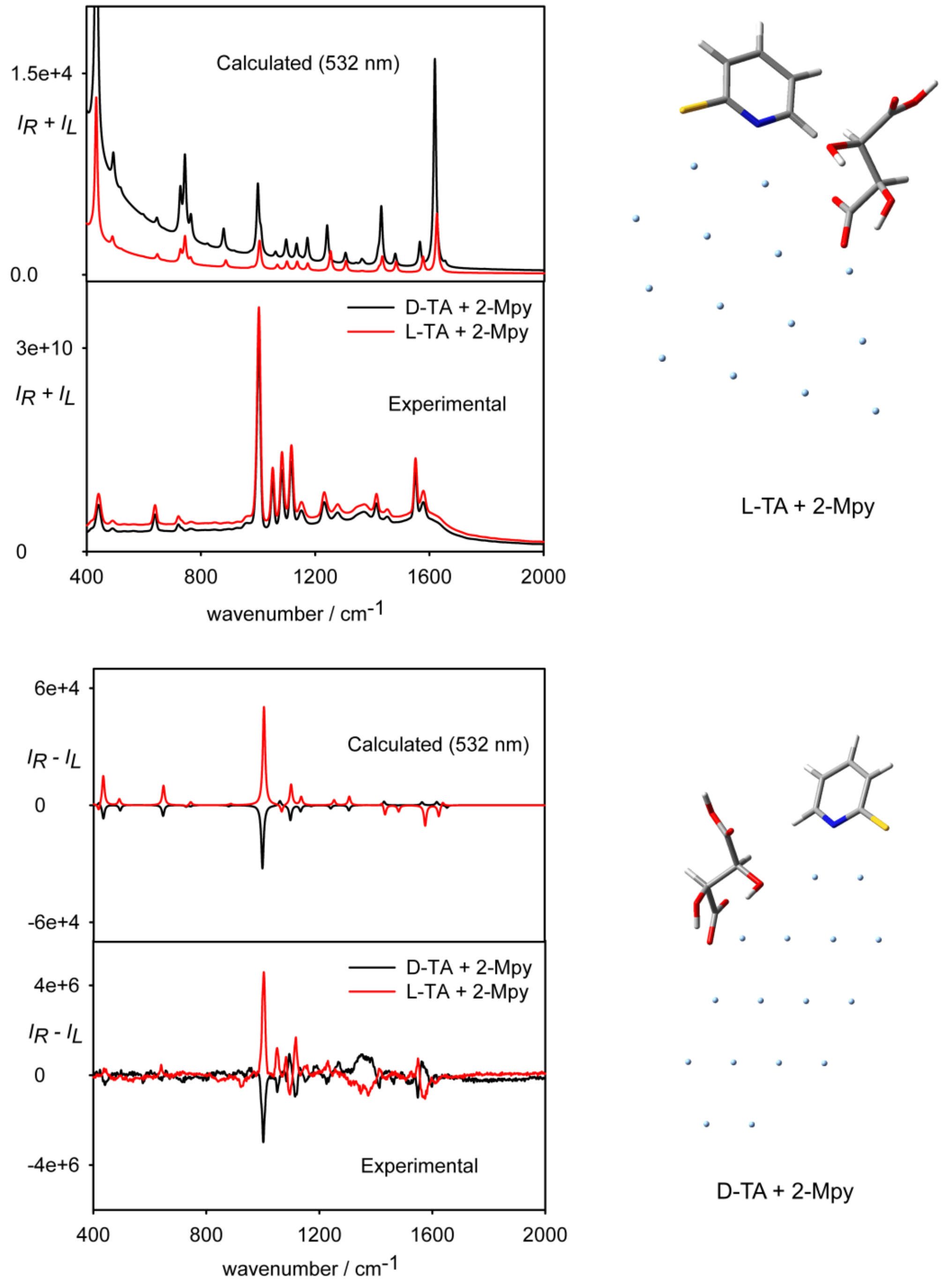

Figure S8. Model of 2-Mpy/ L-, D-TA/ silver cluster of 16 silver atoms. Calculated SEROA spectra at 532 nm excitations show close resemblance to the bisignate experimental SEROA spectra, measured with 532 nm excitation.

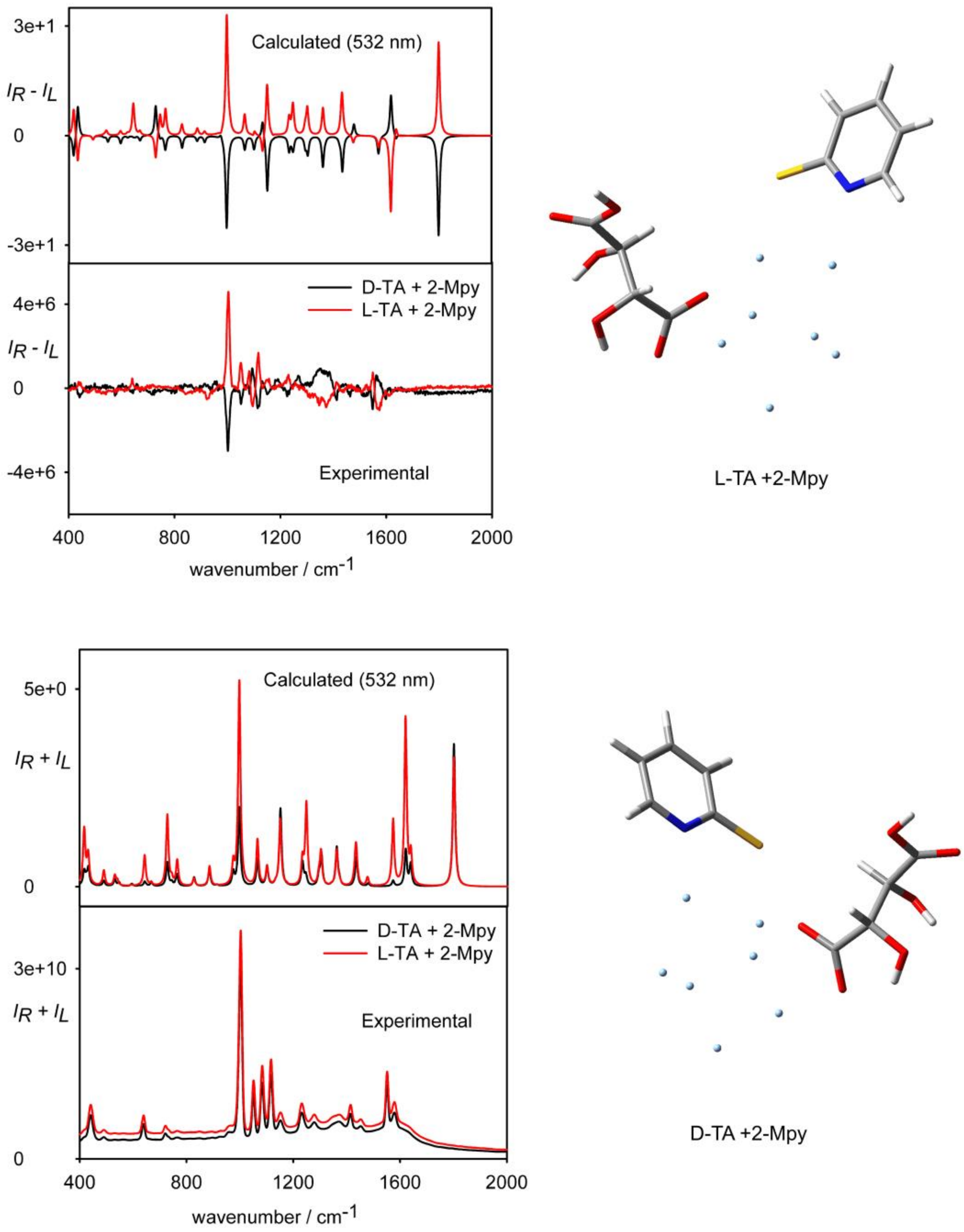

<u>Figure S9</u>. Model of 2-Mpy/ L-, D-TA / silver cluster of 7 silver atoms. Calculated SEROA spectra at 532 nm and 534 nm excitations show close resemblance to the bisignate experimental SEROA spectra, measured with 532 nm excitation.

<u>Table S1.</u> Vibrational assignment of selected SERS bands of TA.

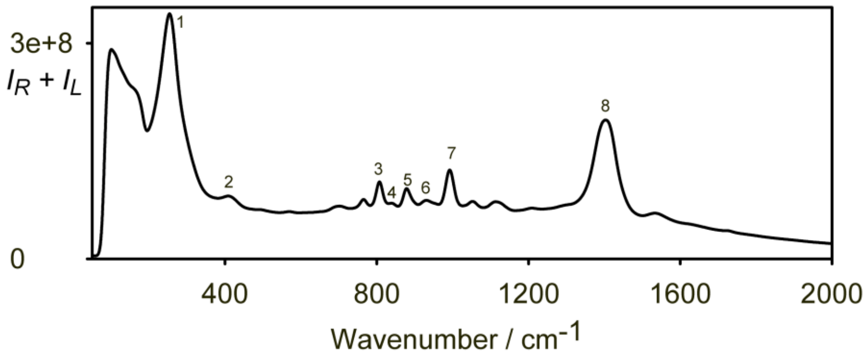

No.	SERS band	Assignment
1	250 cm^{-1}	ν(Ag–TA)
2	410 cm^{-1}	δ(C–C–C)
3, 4	843 cm^{-1}, 806 cm^{-1}	δ(O=C=O)
5, 6	930 cm^{-1}, 880 cm^{-1}	ν(C–COO^-)
7	992 cm^{-1}	ν(C–C)
8	1400 cm^{-1}	ν_s(O=C=O)

ν = stretching; ν_s = symmetric stretching; δ = bending.

Table S2. Vibrational assignment of Cys SERS bands.

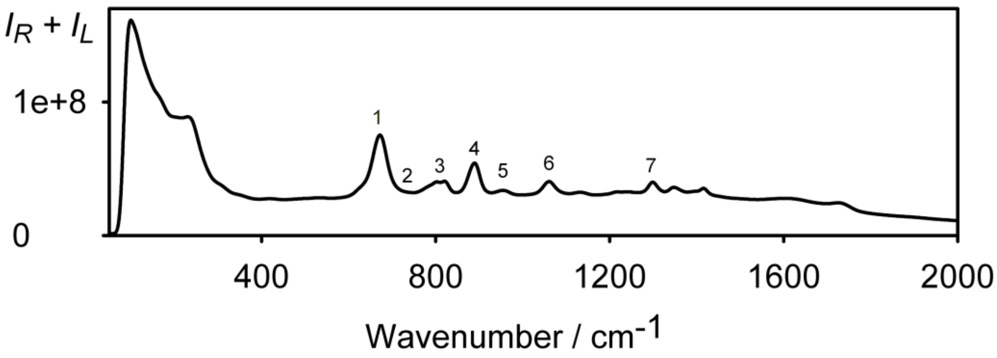

No.	SERS band	Assignment
1	663 cm^{-1}	ν(C–S)
2	725 cm^{-1}	ν(C–S)
3	815 cm^{-1}	δ(H–C–S)
4	890 cm^{-1}	ν(C–COO^-)
5	947 cm^{-1}	δ(H–C–H) + δ(N–C–H)
6	1058 cm^{-1}	ν(C–N)
7	1291 cm^{-1}	δ(C–C–H) δ(H–C–H)

ν = stretching; δ = bending

Table S3. Vibrational assignment of 2-Mpy SERS bands.

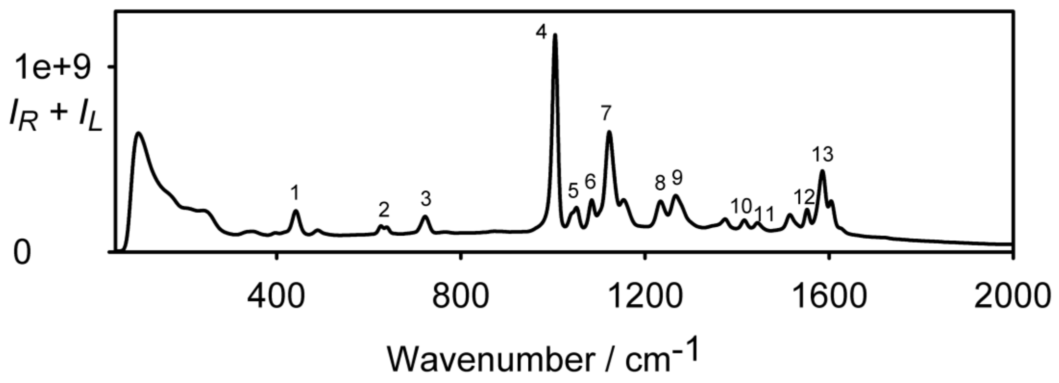

No.	SERS band	Assignment
1	441 cm^{-1}	δ(C=N)$_{oop}$, δ(C–H)$_{oop}$
2	639 cm^{-1}	δ(C=N)$_{oop}$, δ(C–H)$_{oop}$
3	720 cm^{-1}	ν(C–S)
4	1001 cm^{-1}	ν(CC)$_{ring}$
5	1050 cm^{-1}	δ(C–H)$_{ip}$
6	1083 cm^{-1}	δ(C–H)$_{ip}$
7	1117 cm^{-1}	δ(C–H)$_{ip}$
8	1230 cm^{-1}	δ(C–H)$_{ip}$
9	1277 cm^{-1}	ν(CC)$_{ring}$/ν(C–S)
10	1415 cm^{-1}	ν(C=C)/ν(C=N)
11	1452 cm^{-1}	ν(C=C)/ν(C=N)
12	1550 cm^{-1}	ν(C–C)/ν(C=N, C–N)
13	1578 cm^{-1}	ν(C–C)

ν = stretching; δ = bending; oop = out of plane; ip = in plane; ring = ring breathing

Table S4. Vibrational assignment of 4-MBA SERS bands.

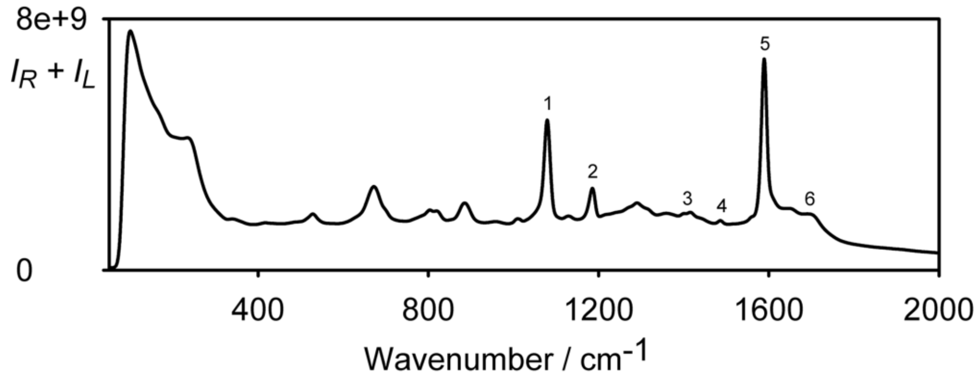

No.	SERS	Assignment
1	1078 cm^{-1}	$\nu(CC)_{ring}$
2	1183 cm^{-1}	$\delta(CH)$
3	1405 cm^{-1}	$\nu_s(COO^-)$
4	1486 cm^{-1}	$\nu(CC) + \delta(CH)$
5	1590 cm^{-1}	$\nu(CC)_{ring}$
6	1698 cm^{-1}	$\nu(CO)$

δ=bending or deformation; ν=stretching; ring = ring breathing.

Table S5. Vibrational assignment of additional SERS bands due to PGA.

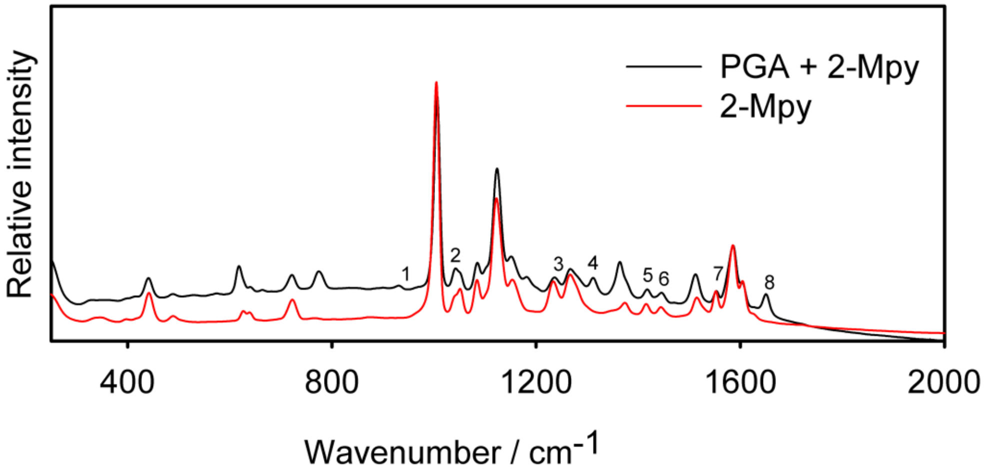

No.	SERS band	Assignment
1	950 cm^{-1}	Localized δ(C–H), out of plane modes
2	1041 cm^{-1}	Wagging and rocking δ(C–H_2).
3, 4	1251 cm^{-1}-1304 cm^{-1}	Amide III
5, 6	1409 cm^{-1}, 1442 cm^{-1}	symmetric δ(CH_2), ν(C–O) and δ(C–H)
7	1563 cm^{-1}	ν_{as}(–CO_2–), Amide II
8	1674 cm^{-1}	Amide I

ν = stretching; δ = bending; ν_{as} = asymmetric stretching.
